# Perinatal Care Provider Perspectives on Integrating Clinical Research Into the Clinical Infrastructure

**DOI:** 10.1111/jmwh.13703

**Published:** 2024-11-12

**Authors:** Yanqiao Li, Kylea L. Liese, Lacey Pezley, Arissara Sawatpanich, Gloria Elam, Katherine Erbe, Lisa Tussing‐Humphreys, Mary Dawn Koenig

**Affiliations:** ^1^ Department of Human Development Nursing Science University of Illinois Chicago Chicago Illinois; ^2^ Department of Kinesiology and Nutrition University of Illinois Chicago Chicago Illinois; ^3^ Ramathibodi School of Nursing, Faculty of Medicine Ramathibodi Hospital Mahidol University Ratchathewi Bangkok Thailand; ^4^ College of Medicine University of Illinois Chicago Chicago Illinois; ^5^ Yvonne L. Munn Center for Nursing Research Massachusetts General Hospital Boston Massachusetts

**Keywords:** clinical research, implementation, pregnancy, system design

## Abstract

**Introduction:**

This study explored perinatal health care providers’ perspectives on the recruitment of pregnant participants and integrating clinical research into their practice, with a particular emphasis on the complexities introduced by the COVID‐19 pandemic.

**Methods:**

From May to September 2021, semistructured interviews were conducted with 10 perinatal health care providers from an urban US health center. The interview transcripts were analyzed using Braun and Clarke's thematic analysis framework, a rigorous method for analyzing qualitative data by identifying, coding, and reporting themes. This approach allowed us to systematically code the data and identify key themes related to recruitment strategies and integration of clinical trials during prenatal care.

**Results:**

Barriers to integrating clinical trials into the perinatal infrastructure included pandemic‐related restrictions, heavy workloads, time constraints, ineffective communication and coordination, and maintaining the relevance of the research among providers. Facilitators included the use of communication tools, collaboration with multidisciplinary teams and stakeholders, creation of detailed study information for clinic staff, and fostering commitment to supporting research among providers.

**Discussion:**

The perspectives of perinatal health care providers uncover barriers and facilitators regarding the recruitment of pregnant individuals for clinical trials and shed light on the unprecedented challenges of research in this population during the COVID‐19 pandemic and lessons learned postpandemic. This information can support the development of evidence‐based solutions and strategies to improve the recruitment of pregnant individuals, as well as enhance clinical research integration into infrastructure in perinatal health clinics.

## INTRODUCTION

Recruitment of participants for research has long been reported as a significant challenge and primary cause of trial delays.^1^ In particular, less than 10% of pregnant people report participation in a research study during their pregnancy.^2^ Previous studies involving pregnant people have reported that only about 20% of their recruitment goals were met during the planned recruitment period.^2^, ^3^ Recruitment of pregnant people for clinical intervention trials is more difficult than other types of research, such as retrospective and observational studies.^2^

The primary reason pregnant people avoid enrolling in clinical research is the perceived risk of harm to the fetus.^4^ Given the length of gestation, study participation for a prolonged period and burdensome intervention protocols can discourage pregnant people from participating.^5^, ^6^ Researchers have often encountered difficulties in recruiting pregnant people belonging to racial and ethnic minorities and low‐income groups due to mistrust of health care providers and personal barriers such as time constraints, transportation limitations, and family obligations.^7^, ^8^, ^9^, ^10^

Although pregnant people express hesitancy about enrolling in clinical research, many report being more likely to enroll if they receive reinforcement regarding the safety of trials from their providers.^11^ Providers can be strong contributors, advocates, and facilitators of the recruitment process and can help researchers integrate research into the clinical infrastructure.^12^ Not only can providers assist the research team in identifying eligible participants,^10^ but providers often establish a trusting relationship with patients that can motivate them to participate. Thus, providers can play a beneficial collaborative role in the research process.^4^

**QUICK POINTS**

✦ Providers can significantly influence recruitment by reinforcing the safety and benefits of trial participation to pregnant individuals.
✦ Provider familiarity and comfort with research details are crucial for effective recruitment and patient trust.
✦ Clinics should implement strategies to reduce research‐related workload for providers to increase their participation in clinical trials.
✦ Researchers should develop effective communication methods to facilitate easier discussion of research opportunities between providers and patients.

Key factors influencing providers’ involvement in recruitment include familiarity with the study, understanding the study's purpose and benefits, knowing their role in the recruitment effort, and fully understanding the eligibility criteria to identify potential participants.^6^, ^11^, ^13^, ^14^, ^15^, ^16^ In fact, many providers see themselves as playing a gatekeeper role for study involvement.^6^, ^10^, ^15^ Providers’ concerns about supporting recruitment included providing study information in an objective way, maintaining patient confidentiality, avoiding coercive language and tactics, and potential negative impacts on the patient‐provider relationship.^6^, ^13^, ^14^, ^17^ Providers are more inclined to participate in recruitment efforts when the study objectives and results might improve their clinical practice and patient outcomes.^11^, ^14^, ^15^, ^18^ Providers’ familiarity and comfort with research are also key to their willingness to help with recruitment, indicating the importance of previous research training.^6^ Clinic time constraints, heavy workload, understaffing, lack of available space, and lack of financial support^6^, ^14^, ^15^, ^16^, ^17^, ^18^, ^19^ impact the provider's ability to assist with study recruitment. Factors important to sustaining provider involvement in research are ongoing communication with the research team, reminders (eg, posters or signposts to avoid forgetfulness and role confusion), support and encouragement from colleagues and organizational leaders, and establishing a research culture within the organization.^6^, ^13^, ^15^, ^16^, ^17^, ^18^, ^19^

Given these factors influencing providers’ recruitment efforts, exploring providers’ perspectives on integrating clinical trial research into the clinical infrastructure is a logical next step. This study explored perinatal health care providers’ perspectives on the barriers and facilitators for integrating clinical trial research into clinical practice. It also examined the challenges and strategies for recruiting pregnant people into a clinical trial involving a daily dietary supplement, all unfolding in the historically noteworthy context of the global COVID‐19 pandemic.

## METHODS

### Participants

We used purposive sampling to recruit perinatal health care providers (certified nurse‐midwives and obstetric physicians) from an urban academic health center between May and September 2021. These perinatal health care providers, who varied in their levels of research experience, were introduced to the study. Those interested consented to participate. Study procedures and materials were approved by the University of Illinois Chicago Institutional Review Board (no. 2020‐1297). The interviewer collected demographic data.

### Data Collection

We conducted individual interviews lasting 60 to 90 minutes. Interviews took place in a room in the labor and delivery unit or via Zoom. All interviews were conducted by an interviewer who identifies as a White cisgender woman, has extensive experience conducting interviews, and had a prior working relationship with the participants. The interviewer asked questions related to the integration of research into the clinical infrastructure, participants’ experiences of working in clinical trials, and their interest and thoughts about their involvement in recruiting for clinical trials (see Table 1). After participants described their experiences, the interviewer asked, “Do you have other thoughts on integrating the clinical trial into the clinical infrastructure?” We posed clarifying questions, such as “Can you tell me more about that?” All prompts were guided by the participant's responses. Transcripts were audio‐recorded and transcribed verbatim.

**Table 1:** Interview Questions

“What kind of involvement do you think providers could have in recruiting participants for the clinical trial?”
“What barrier do you anticipate arising for providers helping with recruitment for the study?”
“Have you been involved in research studies as a participant or as a recruiter?”
“What are your thoughts about the main study?”
“What do you think would work best for recruitment given the workflow of the clinic?”
“How and when would you want the research team to communicate with you about a participant's health status?”

### Data Analysis

Our team worked together to reflect on the analytic process and reach a consensus through investigator triangulation. We followed the Standards for Reporting Qualitative Research (SRQR)^20^ and Consolidated Criteria for Reporting Qualitative Research (COREQ)^21^ to guide our writing. The SRQR and COREQ checklists are available in Supporting Information: Appendix S1 and S2.

Our data analysis was guided by Braun and Clarke's thematic analysis framework. We chose thematic analysis to develop more implicit themes and patterns from the data.^22^ This thematic analysis^22^ was conducted by a research team consisting of a doctoral student and faculty experts in maternal health. Other members of our team include individuals who identify as Black cisgender women, White cisgender women, and Asian cisgender women, all with experience in maternal health care and/or research. Regarding potential biases, we recognized how our own experiences of working as providers and researchers may influence the way we understand and interpret participants’ experiences. Furthermore, we discussed how our experiences working with research participants may impact our interest in specific research questions, guiding theory, and analysis. Therefore, we were careful not to make assumptions or draw conclusions about participants’ experiences informed by prior work.

The first phase of this analysis included rereading the focus group transcripts. The data were individually coded by 3 team members to generate initial codes. The team met to discuss discrepancies and reached a consensus on final codes for the development of themes. Themes represented the experiences of multiple participants. To ensure the validity of our analysis, the team allowed discussion of the analysis, authenticity of coding, and thematic development. Exemplar quotes for each theme are provided in the format of (provider [P] number, line numbers).

### Research Trustworthiness

Our analysis met the criteria of trustworthiness, including credibility, transferability, and confirmability.^23^ Credibility was ensured through prolonged engagement with the data (ie, multiple readings of transcripts, discussions of meaning, context, and quote selection),^23^ peer debriefing (ie, sharing and revising analysis and selection of quotes with research team members who were not involved in the coding process and experts from various health‐related disciplines [eg, nursing, midwifery, anthropology]), and working within a research team. A sufficient description of the methodology and contextualized narratives of participants supported the transferability of the findings. We demonstrated confirmability through discussion of potential biases, as well as using peer debriefing and working within a research team to ensure the use of a data‐oriented approach and an accurate presentation of the data. We also ensured confirmability through the selection of various quotes that were representative of the data and multiple participants’ experiences.

## RESULTS

Our sample was composed of 10 perinatal health care providers (4 obstetricians, 4 certified nurse‐midwives, 1 registered nurse, and 1 clinical director in an administrative role) between 27 and 58 years of age. All identified as female and non‐Hispanic and were employed full‐time (Table 2). The interview focused on identifying providers’ perspectives on the integration of clinical research into the clinical infrastructure, with specific attention to challenges posed by the COVID‐19 pandemic. Two main themes were identified—barriers and facilitators—and within each theme, subthemes emerged.

**Table 2:** Demographics

Characteristic	Value
**Age, y**	
Range	27‐58
Mean (SD)	39.7 (10.18)
Median (IQR)	39.0 (30‐47)
**Race, n (%)**	
Asian	3 (30)
White	4 (40)
Black	2 (20)
Other	1 (10)
**Degree, n (%)**	
MS	4 (40)
MSHA	1 (10)
MD	2 (20)
MD, MPH	2 (20)
DNP	1 (10)
**Practice at site, y**	
Range	0.25‐12
Mean (SD)	3.88 (3.58)
Median (IQR)	3.25 (1‐6)
Total practice, y	
Range	1‐27
Mean (SD)	9.6 (9.4)
Median (IQR)	5 (3‐16)
**Research experience, n (%)**	
None	5 (50)
Limited	5 (50)

Abbreviations: DNP, doctor of nursing practice; IQR, interquartile range; MD, doctor of medicine; MPH, master of public health; MS, master of science; MSHA, master of science in health administration.

### Barriers

#### Heavy Workload and Time Constraints

Providers reported the fast‐paced clinical environment and high patient turnover as barriers to clinic recruitment. As one provider noted, “I think that's just our biggest issue in clinics in general cause they're busy, and a lot of patients are moving ” (P3, lines 85‐86). In addition, providers noted that the burden of the heavy clinical workload could hinder their ability to devote sufficient time and effort to participant recruitment and retention: “I also think that there might be concerns for people, especially those with higher clinical loads, about how much bandwidth they have to help with retention, which, I think, takes some work” (P1, lines 28‐30).

Providers also expressed concern about the prospect of balancing clinical workload with potential disruptions due to recruitment efforts. They reported that the recruitment process can be time‐consuming and disruptive, especially in high‐volume clinics. Providers stressed the importance of minimizing the impact of recruitment on their workflow:

I feel like oftentimes our clinics are overwhelming for some… and we're often double‐booked… So, I think it's gonna be more of how you are going to protect my time and how this is going to make it less of a burden for me and clinic. (P8, lines 215‐220)

High workloads without protected patient time in the clinical infrastructure created obstacles to the recruitment process, as providers had little capacity and space to engage in recruitment activities.

#### Lack of Effective Communication and Coordination

Providers stressed the importance of clear communication and coordination with the research team to ensure seamless integration with clinic staff and minimal disruption to clinic operations:

Just communicate beforehand, “Hey, what's your workflow? Can I go in there now, or do you want me to wait? Or should I go in the other room first?” Just communicate with the provider. And some people, you know, get more worked up than others, no matter what you do. (P6, lines 152‐155)

To ensure that the clinic functioned smoothly and with minimal disruption to providers, effective communication and coordination between the research team members and clinic staff was imperative.

#### Unable to Sustain the Salience of Study

Perinatal health care providers had to balance competing priorities and tasks during their clinic days. Consequently, maintaining the relevance of research to providers was critical. Providers highlighted the importance of collaboration and the need for reminders throughout the study duration to ensure that the research project remained a priority:

I really think the main one is just like keeping it salient. We have that urine study going on, and it's been going on for a while but, when it drops out of your radar you just forget about it and then I'm sure we missed a ton of recruitment opportunities because we just forgot about it, cause there's so much other stuff to do… so there just need to be a way to keep it at the forefront. (P1, lines 85‐90)

Considering the multiple studies being conducted in the clinic at one time, providers reinforced the need for consistent and salient reminders to ensure the research in question remained prominent in their minds. Lacking such reminders, studies will naturally fade into the background, forgotten by busy providers and thereby missing out on potential participants.

#### Impact of Social Distancing and Recruitment Efforts During the COVID‐19 Pandemic

In response to the increased transmissibility of COVID‐19, hospitals and clinics implemented safety measures to prevent transmission and exposure. However, these measures (including visitor restrictions and limitations on clinic occupancy) created barriers to research recruitment. Providers reported difficulties complying with restrictions, particularly when multiple study team members attended the clinic to recruit patients. This resulted in space constraints for clinic staff and subsequent recruitment challenges for study teams. One provider described difficulties in balancing safety policies with patient recruitment:

It's challenging when there's a study going on, and there are multiple individuals participating in the recruitment efforts of that study. It just kind of can crowd out the clinic, you know, we've had some recruitment efforts, where, you know, you have the study the principal investigator, maybe in the clinic and some research assistants that are trying to recruit standing in the hallways. (P9, lines 160‐165)

Providers reported that they were concerned about adherence with COVID‐19 safety policies and maintaining social distancing in small clinic spaces, while simultaneously balancing the needs of research teams, students, and other staff. These social distancing concerns continued postpandemic:

So, I think lessons learned and at least from what I've heard about with COVID, it is making sure that we're limiting the amount of people coming in at least from a provider and researchers’ standpoint to be with patients. We want to make sure that we are still social distancing. We've had time for researchers to come in with larger teams and then that's really challenging because we're saying that we can't have specific learners in some location and then there is a group of 12 people walking together. (P10, lines 195‐201)

Providers underscored the importance of adapting recruitment strategies to meet the clinic pandemic and postpandemic guidelines. There was a need to prioritize adherence with safety policies and limit the number of study team members to ensure social distancing within the clinic was maintained.

### Facilitators

#### Communication Through Electronic Health Records

Providers expressed interest and enthusiasm for the use of electronic health record technology as a communication tool between the provider and research team members. As one provider explained, “It is golden for us because we're very limited in space. So, you know if you can do a lot of it that way, it will be helpful” (P9, lines 250‐252). Providers identified the electronic health record as a way to be informed of upcoming recruitment opportunities and potentially eligible patients. In addition, for managing already enrolled participants, one provider suggested implementing reminders for critical patient information:

I would like it to be flagged in my next visit… Like as a sticky note, or something does pop up to say like: “Hey, your patient in the study, and her hemoglobin is 8. So, I want to make sure that you are aware…”(P7, lines 337‐342)

Sending reminders through the communication feature in the electronic health record was a helpful strategy. As one provider pointed out, “Maybe you guys come up with a MyChart message that just sends out a reminder every month, okay just remember get your blood drawn today” (P5, lines 126‐128).

Providers identified the integration of technology with electronic health records as an effective tool for communication, which could facilitate the coordination of research tasks with their schedules.

#### Collaborative Partnerships and Key Stakeholder Support

Partnering with multidisciplinary teams and securing their support facilitated the recruitment process. Providers recommended involving *practice representatives* who were familiar with clinic flow (typically with expertise in perinatal health clinic visits) to assist the study team in approaching patients for recruitment. One provider mentioned the value of having a liaison within the clinics who represents the practices:

I think it would be ideal if you have a contact person in the clinics that are representative of practices; for example, if it is like a midwife or a physician at the Center for Women's Health… I think having a collaborative relationship in engaging with them in the last part of the IRB would help. I think having them involved if there is like a kickoff meeting for the project to have them informed with a presentation would be very useful. (P7, lines 172‐178)

Providers recommended involving key stakeholders, people with a direct stake and important role in the clinic's success (such as clinic administrators or medical assistants), to facilitate the successful implementation of the study in the clinic. Gaining support and collaboration from stakeholders could lead to a smoother recruitment process:

I think if we get a good buy‐in if we really describe the patient flow, I think we have the right infrastructure. I believe you should be able to do that in it's a very much needed study because we are really struggling in it. (P7, lines 383‐386)

Providers reported clinic staff and providers could play a pivotal role as key stakeholders in the implementation of a successful research study. Through actively engaging them from the outset and fostering collaborative partnerships, research could be conducted with greater efficiency and productivity within the clinical infrastructure.

#### Empowering Interviews and Enhancing Efficiency With Written Scripts

Providers reported feeling unsure of how to introduce studies and how to highlight the importance of participating in the research project. Providers proposed creating a comprehensive written script addressing questions frequently asked by patients, which could improve recruitment productivity among the providers. Such a written script would allow the provider to respond appropriately to inquiries during the visit:

I know you'll have the consent as well, but even just something like whatever like a written script rather than a verbal script for the consent process might be helpful as well, so that way, the residents can say you know, do you have any questions about this project that you're being asked to take a part in and if they do, then the resident can answer those. (P8, lines 275‐280)

Providers believed that a written script would not only ensure clarity and consistency in the information provided to potential participants but also empower providers to address most queries related to the research study.

#### Sense of Obligation and Commitment to Support Research Studies

Providers felt a sense of responsibility and duty to support research that could bring benefits to their patients. They recognized the value of clinical research contributing to evidence‐based practices. One provider shared a comment from another staff member in the clinic about this issue: “We don't do enough of this type of research to put [the university] on the map… I support you 100%, and I will help get the nurses involved in these things” (P8, lines 382‐385). Another provider also recognized that facilitating research was part of the responsibility of practicing at an academic institution: “We are at an academic institution, and [if] it sounds like something that, like they could help a lot of our patient population, then I think most of us are happy to be a part of it.” (P1, lines 98‐100).

Providers exhibited a strong sense of responsibility to support research benefiting their patients, which was reinforced by their clinic's affiliation with an academic institution. Emphasizing the clinic's academic affiliation and its responsibilities to support research and practice improvement was crucial to the integration of research into the clinical infrastructure.

## DISCUSSION

We are among the first to study the perspectives of perinatal health care providers regarding the integration of research into the clinical infrastructure of a perinatal health clinic.

The data reveal significant barriers and facilitators associated with conducting research in a perinatal health clinic setting and provide a broad understanding of the complexities involved in the recruitment of pregnant people within a clinical setting. Clinic recruitment can be hampered by a variety of barriers, including public health‐related restrictions, heavy workloads, time constraints, and communication issues. On the other hand, recruitment can be facilitated by effective and frequent communication, collaboration with staff and providers, and by nurturing a sense of obligation and commitment to research among providers. These facilitators suggest potential strategies for overcoming challenges and optimizing the recruitment process. These findings emphasize the need for strategic approaches to research recruitment that consider both the unique challenges of the perinatal health environment and the broader impact of both the COVID‐19 pandemic and postpandemic challenges on both research and clinic operations.

Heavy workloads and time constraints are significant barriers to active participation in clinical research. Our work is consistent with existing literature emphasizing that burdensome clinic schedules and workloads pose formidable challenges to providers’ participation in clinical research.^6^, ^14^, ^17^, ^19^, ^24^, ^25^ In a study among midwives, their willingness to facilitate the research process was contingent on the study's complexity, its burdensome nature, and the time required to explain the study to their patients.^26^ Notably, providers consistently prioritize their duty of care over research recruitment.^15^, ^26^ Adding recruitment responsibilities to an already heavy workload, coupled with differing attitudes and perspectives on research among providers, researchers, and administrators, may lead them to perceive research as an additional burden.^27^ In one study, it was found that the burden of the clinical research process was the sole significant factor influencing physicians’ decisions to refer patients to a clinical trial, suggesting that physicians were primarily concerned with the demands of the trial when considering patient referrals.^28^ These findings emphasize the need for strategies that take into account the workload and time constraints of providers to ensure that integrating research into clinical practice is feasible and sustainable.

The dynamic clinical environment presents a challenge in maintaining the prominence of a specific study, particularly when multiple trials are conducted concurrently. As identified in previous research, such competition can contribute to the failure of studies due to the struggle for attention.^29^ Moreover, limited communication between providers and researchers can lead to decreased trial awareness, thereby limiting providers’ ability to inform patients about potential participation opportunities. However, providers can play a crucial role in study enrollment. Effective communication between providers and the research team can significantly streamline the recruitment process and enhance overall clinic efficiency.^9^ Furthermore, providing training and education about clinical research to providers is a critical step in facilitating recruitment.^13^ As presented in our study, recruitment tools such as written scripts and specific communication tools can help alleviate some of the communication burden related to clinical research. Supporting this, the use of interpersonal communication, the use of print materials, and unified messaging among clinical staff enhanced the willingness of pregnant individuals to participate in clinical research.^9^, ^17^, ^19^ Efficient communication channels and adequate preparation of providers on how to introduce the trial to patients are essential for them to feel adequately prepared for recruitment tasks.^19^

Challenges imposed by the COVID‐19 pandemic highlight the preexisting inefficiencies of integrating clinical research into the clinical infrastructure of a perinatal health clinic. In fact, certain pandemic policies (such as visitor restrictions and the use of telemedicine) have continued postpandemic. These policies create challenges for researchers but should be considered as an opportunity to promote dialogue and problem‐solving among researchers and clinic stakeholders on the reforms needed to integrate research into the clinic infrastructure postpandemic.^30^ The number of physical visits to the clinic postpandemic is reduced with the introduction of telemedicine. This is a direct result of the COVID‐19 pandemic and provides an opportunity for the research enterprise to adapt with the creation of novel trial designs,^30^ including online consent processes and online data collection methods. Lessons learned from the COVID‐19 pandemic can be applied postpandemic to streamline the integration of research into clinical settings while still maintaining methodologically robust designs.

Our interviewees demonstrated a strong sense of commitment and responsibility to research. This aligns with previous research, which found that a provider's professional identity and involvement in clinical research is a significant predictor of their intention to support clinical research.^24^ Furthermore,^30^ our study revealed that providers are more likely to actively participate in a study if they find its aims interesting and perceive potential benefits for their patients.^6^, ^15^, ^24^, ^26^ They are more comfortable and willing to recruit for trials that they believe patients would find acceptable. Our findings also mirror those of previous studies, which suggest that the clinic's culture and environment can influence providers’ willingness and motivation to support research.^6^ Our study contributes to the existing body of knowledge by emphasizing the role of provider commitment, the influence of trial alignment with professional opinions, and the impact of clinic culture on research participation.

### Limitations

Although our study provides valuable insights into the experiences of perinatal health care providers participating in clinical research, several limitations should be noted. First, our findings are derived from a small sample of providers at a single urban academic health center, which may constrain the generalizability of our results to other settings or regions. Second, the study was conducted during the COVID‐19 pandemic, a unique context that may have shaped the experiences and perspectives of the providers. Consequently, the applicability of our findings to postpandemic circumstances may be limited. Third, our study relied exclusively on interview data. Additional methods, such as observation or document analysis, could have offered a deeper understanding of the integration of research into a clinical setting. Despite these limitations, our study is unique in contributing to understanding provider perspectives on integrating clinical research into the clinical infrastructure, particularly during a pandemic.

## CONCLUSION

This study provides valuable insights into the perspectives of perinatal health care providers on the integration of research into the clinical infrastructure. The impact of the COVID‐19 pandemic, heavy workload, and time constraints emerged as significant barriers that perinatal health care providers face in recruiting for clinical trials. Effective communication tools and strategies were identified as facilitators that can alleviate some of the challenges and enhance recruitment efforts by providers. Moreover, providers’ sense of obligation and commitment, influenced by their academic affiliation, along with the prevailing culture in the clinic, played a crucial role in their willingness to assist with clinical trials. By recognizing and addressing these barriers, researchers and health care institutions can enhance the integration of clinical research and improve the recruitment of pregnant individuals. Further research is warranted to explore additional strategies and interventions to overcome these barriers. Additionally, research is needed to develop supportive policies and practices that foster a culture of support for research within the clinic.

## CONFLICT OF INTEREST

All authors have no conflicts of interest to declare.

## Supporting information




**Appendix S1**. Standards for Reporting Qualitative Research (SRQR) Checklist

**Appendix S2**. Consolidated Criteria for Reporting Qualitative Research (COREQ) Checklist

## References

[1] Giffin RB , Lebovitz Y , English RA . Transforming Clinical Research in the United States: Challenges and Opportunities: Workshop Summary. National Academies Press; 2010. 21210556

[2] Sutton EF , Cain LE , Vallo PM , Redman LM . Strategies for successful recruitment of pregnant patients into clinical trials. Obstet Gynecol. 2017;129(3):554‐559. 28178062 10.1097/AOG.0000000000001900 PMC5321786

[3] van Delft K , Schwertner‐Tiepelmann N , Thakar R , Sultan A . Recruitment of pregnant women in research. J Obstet Gynaecol. 2013;33(5):442‐446. 23815192 10.3109/01443615.2013.767787

[4] Blehar MC , Spong C , Grady C , Goldkind SF , Sahin L , Clayton JA . Enrolling pregnant women: issues in clinical research. Womens Health Issues. 2013;23(1):e39‐e45. doi:10.1016/j.whi.2012.10.003 23312713 PMC3547525

[5] Oude Rengerink K , Logtenberg S , Hooft L , Bossuyt PM , Mol BW . Pregnant womens’ concerns when invited to a randomized trial: a qualitative case control study. BMC Pregnancy Childbirth. 2015;15(1):207. 26341516 10.1186/s12884-015-0641-x PMC4560072

[6] Tooher RL , Middleton PF , Crowther CA . A thematic analysis of factors influencing recruitment to maternal and perinatal trials. BMC Pregnancy Childbirth. 2008;8:36. doi:10.1186/1471-2393-8-36 18687110 PMC2532678

[7] Rodrigues Amorim Adegboye A , Cocate PG , Benaim C , et al. Recruitment of low‐income pregnant women into a dietary and dental care intervention: lessons from a feasibility trial. Trials. 2020;21(1):244. 32138765 10.1186/s13063-020-4142-5 PMC7057473

[8] Andrews L , Davies TH . Participant recruitment and retention from vulnerable populations in clinical trials is a matter of trust. Contemp Clin Trials. 2022;123:106969. 36273802 10.1016/j.cct.2022.106969

[9] Frew PM , Saint‐Victor DS , Isaacs MB , et al. Recruitment and retention of pregnant women into clinical research trials: an overview of challenges, facilitators, and best practices. Clin Infect Dis. 2014;59(suppl 7):S400‐S407. 25425718 10.1093/cid/ciu726 PMC4303058

[10] van der Zande ISE , van der Graaf R , Oudijk MA , van Vliet‐Lachotzki EH , van Delden JJM . A qualitative study on stakeholders’ views on the participation of pregnant women in the APOSTEL VI study: a low‐risk obstetrical RCT. BMC Pregnancy Childbirth. 2019;19(1):65. doi:10.1186/s12884-019-2209-7 30744577 PMC6371564

[11] Wada K , Evans MK , de Vrijer B , Nisker J . Clinical research with pregnant women: perspectives of pregnant women, health care providers, and researchers. Qual Health Res. 2018;28(13):2033‐2047. doi:10.1177/1049732318773724 29865990

[12] Frew PM , Saint‐Victor DS , Isaacs MB , et al. Recruitment and retention of pregnant women into clinical research trials: an overview of challenges, facilitators, and best practices. Clin Infect Dis. 2014;59(Suppl 7):S400‐S407. doi:10.1093/cid/ciu726 25425718 PMC4303058

[13] Malm MC , Hesselman S , Brismar Wendel S , Ericson J . Episiotomy or not? Midwives’ experiences of recruiting to a randomized controlled trial during pregnancy and labor ‐ a qualitative study. Sex Reprod Healthc. 2021;30:100674. doi:10.1016/j.srhc.2021.100674 34741843

[14] Rose J , Lynn K , Akister J , Maxton F , Redsell SA . Community midwives’ and health visitors’ experiences of research recruitment: a qualitative exploration using the theoretical domains framework. Prim Health Care Res Dev. 2021;22:e5. doi:10.1017/s1463423621000050 33509327 PMC8057511

[15] Hanrahan V , Gillies K , Biesty L . Recruiters’ perspectives of recruiting women during pregnancy and childbirth to clinical trials: a qualitative evidence synthesis. PLoS One. 2020;15(6):e0234783. doi:10.1371/journal.pone.0234783 32559236 PMC7304625

[16] Halkoaho A , Vähäkangas K , Häggman‐Laitila A , Pietilä AM . Views of midwives about ethical aspects of participation in placental perfusion studies. Midwifery. 2012;28(1):131‐137. doi:10.1016/j.midw.2011.02.003 21459500

[17] Mudd LM , Pham X , Nechuta S , Elliott MR , Lepkowski JM , Paneth N . Prenatal care and delivery room staff attitudes toward research and the National Children's Study. Matern Child Health J. 2008;12(6):684‐691. doi:10.1007/s10995-008-0393-6 18668359

[18] Hanrahan V , Lawrie L , Duncan E , Biesty L , Gillies K . Development of a co‐designed behaviour change intervention aimed at healthcare professionals recruiting to clinical trials in maternity care. Trials. 2022;23(1):870. doi:10.1186/s13063-022-06816-6 36224619 PMC9556136

[19] Krebs F , Lorenz L , Nawabi F , et al. Recruitment in health services research‐a study on facilitators and barriers for the recruitment of community‐based healthcare providers. Int J Environ Res Public Health. 2021;18(19):10521. doi:10.3390/ijerph181910521 34639820 PMC8508262

[20] O'Brien BC , Harris IB , Beckman TJ , Reed DA , Cook DA . Standards for reporting qualitative research: a synthesis of recommendations. Acad Med. 2014;89(9):1245‐1251. doi:10.1097/acm.0000000000000388 24979285

[21] Tong A , Sainsbury P , Craig J . Consolidated criteria for reporting qualitative research (COREQ): a 32‐item checklist for interviews and focus groups. Int J Qual Health Care. 2007;19(6):349‐357. doi:10.1093/intqhc/mzm042 17872937

[22] Braun V , Clarke V . Using thematic analysis in psychology. Qual Res Psychol. 2006;3(2):77‐101. doi:10.1191/1478088706qp063oa

[23] Lincoln YS , Guba E . Naturalistic Inquiry. Sage Publications; 1985.

[24] Millar MM , Taft T , Weir CR . Clinical trial recruitment in primary care: exploratory factor analysis of a questionnaire to measure barriers and facilitators to primary care providers’ involvement. BMC Prim Care. 2022;23(1):311. doi:10.1186/s12875-022-01898-2 36463123 PMC9719201

[25] Stock CJ , Carley N , Hickman B , et al. Clinician engagement is critical to public engagement with clinical trials. BMJ. 2015;350:h3140. doi:10.1136/bmj.h3140 26071322

[26] Daly D , Hannon S , Brady V . Motivators and challenges to research recruitment ‐ a qualitative study with midwives. Midwifery. 2019;74:14‐20. doi:10.1016/j.midw.2019.03.011 30925414

[27] Sullivan‐Bolyai S , Bova C , Deatrick JA , et al. Barriers and strategies for recruiting study participants in clinical settings. West J Nurs Res. 2007;29(4):486‐500. doi:10.1177/0193945907299658 17538128

[28] Ramirez AG , Chalela P , Suarez L , et al. Early phase clinical trials: referral barriers and promoters among physicians. J Community Med Health Educ. 2012;2(8):1000173. 24073358 PMC3782313

[29] Briel M , Olu KK , von Elm E , et al. A systematic review of discontinued trials suggested that most reasons for recruitment failure were preventable. J Clin Epidemiol. 2016;80:8‐15. doi:10.1016/j.jclinepi.2016.07.016 27498376

[30] Castelo‐Branco L , Awada A , Pentheroudakis G , et al. Beyond the lessons learned from the COVID‐19 pandemic: opportunities to optimize clinical trial implementation in oncology. ESMO Open. 2021;6(5):100237. doi:10.1016/j.esmoop.2021.100237 34411971 PMC8302832

