# Supplementary material for: Perinatal Care Provider Perspectives on Integrating Clinical Research Into the Clinical Infrastructure
Source: J Midwifery Womens Health. 2024 Nov 12;70(2):301–7. doi: 10.1111/jmwh.13703 (PMC11980763; doi:10.1111/jmwh.13703)
Supplement: Supplementary file 2 — Appendix S2. Consolidated Criteria for Reporting Qualitative Research (COREQ) Checklist [file JMWH-70-301-s001.docx]

**Appendix S2.** Consolidated criteria for reporting qualitative research (COREQ): a 32-item checklist

| Item No. | Topic | Guide question/Description | Reported on Page No./Section |
| --- | --- | --- | --- |
| Domain 1: Research Team and Reflexivity | | | |
| Personal Characteristics | | | |
| 1. | Interviewer/facilitator | Which author/s conducted the interview or focus group? | p. 4 Methods (Data Collection) |
| 2. | Credentials | What were the researcher’s credentials? E.g. PhD, MD | Title page |
| 3. | Occupation | What was their occupation at the time of the study? | p. 4 Methods (Data Analysis) |
| 4. | Gender | Was the researcher male or female? | p. 4 Methods (Data Analysis) |
| 5. | Experience and training relationship with participants | What experience or training did the researcher have? | p. 4 Methods (Data Analysis) |
| 6. | Relationship established | Was a relationship established prior to study commencement? | p. 4 Methods (Data Analysis) |
| 7. | Participant knowledge of the interviewer | What did the participants know about the researcher? e.g. personal goals, reasons for doing the research | p. 4 Methods (Data Collection) |
| 8. | Interviewer characteristics | What characteristics were reported about the interviewer/facilitator? e.g. Bias, assumptions, reasons and interests in the research topic | p. 4 Methods (Data Analysis) |
| Domain 2: Study Design | | | |
| Theoretical Framework | | | |
| 9. | Methodological orientation and theory | What methodological orientation was stated to underpin the study? e.g. grounded theory, discourse analysis, ethnography, phenomenology, content analysis | p. 4 Methods (Data Analysis) |
| Participant Selection | | | |
| 10. | Sampling | How were participants selected? e.g. purposive, convenience, consecutive, snowball | p. 3 Methods (Participants) |
| 11. | Method of approach | How were participants approached? e.g. face-to-face, telephone, mail, email | p. 4 Methods (Data Collection) |
| 12. | Sample size | How many participants were in the study? | p. 5 Results |
| 13. | Non-participation | How many people refused to participate or dropped out? Reasons? | NR |
| Setting | | | |
| 14. | Setting of data collection | Where was the data collected? e.g. home, clinic, workplace | p. 4 Methods (Data Collection) |
| 15. | Presence of non-participants | Was anyone else present besides the participants and researchers? | p. 4 Methods (Data Collection) |
| 16. | Description of sample | What are the important characteristics of the sample? e.g. demographic data, date | p. 5 Results & Table 2 |
| Data Collection | | | |
| 17. | Interview guide | Were questions, prompts, guides provided by the authors? Was it pilot tested? | p. 4 Methods (Data Collection) |
| 18. | Repeat interviews | Were repeat interviews carried out? If yes, how many? | p. 4 Methods (Data Collection) |
| 19. | Audio/visual recording | Did the research use audio or visual recording to collect the data? | p. 4 Methods (Data Collection) |
| 20. | Field notes | Were field notes made during and/or after the interview or focus group? | NR |
| 21. | Duration | What was the duration of the interviews or focus group? | p. 4 Methods (Data Collection) |
| 22. | Data saturation | Was data saturation discussed? | NR |
| 23. | Transcripts returned | Were transcripts returned to participants for comment and/or correction? | NR |
| Domain 3: Analysis and Findings | | | |
| Data Analysis | | | |
| 24. | Number of data coders | How many data coders coded the data? | p. 4 Methods (Data Analysis) |
| 25. | Description of the coding tree | Did authors provide a description of the coding tree? | NR |
| 26. | Derivation of themes | Were themes identified in advance or derived from the data? | p. 4 Methods (Data Analysis) |
| 27. | Software | What software, if applicable, was used to manage the data? | NR |
| 28. | Participant checking | Did participants provide feedback on the findings? | NR |
| Reporting | | | |
| 29. | Quotations presented | Were participant quotations presented to illustrate the themes / findings? Was each quotation identified? e.g. participant number | p. 4 Methods (Data Analysis) |
| 30. | Data and findings consistent | Was there consistency between the data presented and the findings? | p. 4 Methods (Data Analysis) |
| 31. | Clarity of major themes | Were major themes clearly presented in the findings? | p. 4 Methods (Data Analysis) & p. 5 Results |
| 32. | Clarity of minor themes | Is there a description of diverse cases or discussion of minor themes? | p. 4 Methods (Data Analysis) & p. 5 Results |

Note: NR (Not reported)
